# A thin multifunctional coating on a separator improves the cyclability and safety of lithium sulfur batteries†
†Electronic supplementary information (ESI) available: Detailed description of the experimental procedures and calculations. See DOI: 10.1039/c7sc01961k


**DOI:** 10.1039/c7sc01961k

**Published:** 2017-07-17

**Authors:** Guiyin Xu, Qing-bo Yan, Shitong Wang, Akihiro Kushima, Peng Bai, Kai Liu, Xiaogang Zhang, Zilong Tang, Ju Li

**Affiliations:** a Jiangsu Key Laboratory of Material and Technology for Energy Conversion , College of Material Science and Engineering , Nanjing University of Aeronautics and Astronautics , Nanjing 210016 , P. R. China . Email: azhangxg@nuaa.edu.cn; b Department of Nuclear Science and Engineering , Massachusetts Institute of Technology , Cambridge , Massachusetts 02139 , USA . Email: liju@mit.edu; c College of Materials Science and Opto-Electronic Technology , University of Chinese Academy of Sciences , Beijing 100049 , P. R. China; d Department of Materials Science and Engineering , Advanced Materials Processing and Analysis Center , University of Central Florida , Orlando , Florida 32826 , USA; e Department of Chemical Engineering , Massachusetts Institute of Technology , Cambridge , Massachusetts 02139 , USA; f Department of Energy , Environmental and Chemical Engineering , Washington University in St. Louis , Saint Louis , Missouri 63130 , USA; g State Key Lab of New Ceramics and Fine Processing , School of Materials Science and Engineering , Tsinghua University , Beijing 100084 , P. R. China; h Department of Materials Science and Engineering , Massachusetts Institute of Technology , Cambridge , Massachusetts 02139 , USA

## Abstract

Lithium–sulfur batteries are one of the most promising next-generation batteries due to their high theoretical specific capacity, but are impeded by the low utilization of insulating sulfur, unstable morphology of the lithium metal anode, and transport of soluble polysulfides. Here, by coating a layer of nano titanium dioxide and carbon black onto a commercial polypropylene separator, we demonstrate a new composite separator that can confine the polysulfides on the cathode side, forming a catholyte chamber, and at the same time block the dendritic lithium on the anode side. Lithium–sulfur batteries using this separator show a high initial capacity of 1206 mA h g^–1^ and a low capacity decay rate of 0.1% per cycle at 0.5C. Analyses reveal the electrocatalytic effect and the excellent dendrite-blocking capability of the ∼7 µm thick coating.

## Abstract 2

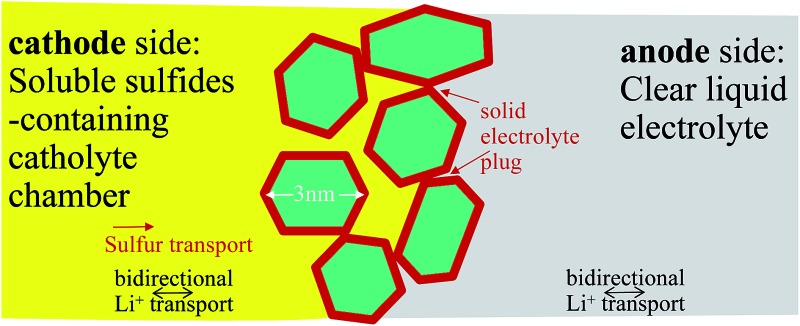
The separator has an electrocatalytic effect for polysulfide transformation, and can confine the polysulfides within the cathode and block the dendritic lithium in the anode.

## Introduction

**Scheme 1:**
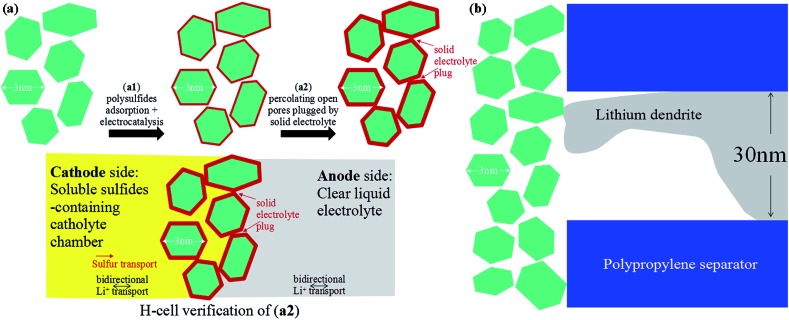
(a) Illustration of two strategies for counteracting polysulfide shuttling in Li–S batteries. (b) Lithium dendrite may find it difficult to plate through few-nm nanopores.

Lithium–sulfur (Li–S) batteries are attractive due to their low cost and high gravimetric energy density,^1^–^3^ but suffer from low cyclability and poor safety in energy-density-optimized full cells due to (a) soluble polysulfide shuttling from the cathode to anode, and (b) lithium metal anode corrosion and shorting.^4^,^5^ In order to defeat (a), there are two strategies (Scheme 1a): (a1) adsorption + electrocatalysis, and (a2) complete sealing by a solid electrolyte. In (a1), the sulfur cathode is mixed with electrocatalyst nanoparticles that compete with the liquid electrolyte for free polysulfides. The electrocatalysts (such as graphene oxide^6^,^7^ and TiO_2_ 8–11) also facilitate the redox reactions of the surface-adsorbed polysulfides. The (a1) route reduces the concentrations and lifetimes of soluble polysulfides, thereby reducing – but not eliminating – sulfur transport to the lithium anode. In the (a2) strategy, one aims to eliminate sulfur crossover completely by sealing off the cathode chamber using a solid electrolyte that conducts Li^+^ but not sulfur.^12^ This is possible because diffusion mechanisms are fundamentally different in solids (“hopping/exchange”) than those in liquids (“vehicular” Stokes–Einstein transport of all soluble species). A solid barrier formed *in situ* with no percolating pores could stop sulfur transport completely while still allowing bidirectional Li^+^ transport, forming an enclosed catholyte chamber on the sulfur side. Moreover, a negatively charged Debye layer near the separator surface could reject the polysulfide anions to localize their transport on the cathode side^13^ and the functional interlayers could also trap lithium polysulfides.^14^,^15^ In order to defeat (b), in particular electrical shorting by dendritic penetration of the separator,^16^ a deformable solid electrolyte separator is also envisioned, which blocks lithium dendrite growth more effectively than traditional nanoporous polypropylene (PP) separators at large current densities.

While lithium metal growth always chases the ionic current and thus the ∼30 nm pores in the PP separator, there can be a thermodynamic cost when the pores get very small. Depending on the over-potential applied to the anode, there is a smallest radius-of-curvature for depositing lithium metal allowed by thermodynamics due to capillary forces.^17^ While lithium metal dendrite is able to plate through the 30 nm pores of the PP separator, it may find it difficult to plate through the much smaller pores of our coating. *In situ* transmission electron microscopy (TEM) observations of mossy lithium growth reveal that even under a very large overpotential,^18^ the smallest mossy lithium tendrils have radii of tens of nanometers, so the fine pores of our ceramic coating could present significant resistance to lithium metal dendrite growth (Scheme 1b), while still allowing bidirectional Li^+^ transport. When this coating is applied without carbon black (*e.g.* to the opposite side of the PP separator), it could add a significant safety factor against electrical shorting.^19^–^21^

In this work, we propose a multifunctional coating less than 10 µm thick, easily applied onto traditional nanoporous PP separators, which addresses (a1), (a2) and (b) simultaneously. This porous coating consists of titanium dioxide nanoparticles and carbon black, forming an excellent adsorbent, and is electronically conductive and electrocatalytically active, verified by shifted redox voltage peaks and theoretical calculations (a1). Later, this thin porous coating gets fouled by solid sulfur-containing compounds, forming an *in situ* solid electrolyte layer that stops sulfur transport while still allowing bidirectional Li^+^ transport. Ideally, if the sulfur-containing solid electrolyte formed *in situ* closes all percolating pores, we will have (a2). To demonstrate this, we applied the fouled coated separator (after cycling) in a H-cell, and showed it can separate the polysulfide containing left side (dark color) with the clear side, demonstrating that it can be used to form a catholyte chamber. Lastly, for (b), we showed that the sub-10 µm thick coating on 30 µm thick PP can delay dendrite penetration for a 15× longer time duration, at an extremely large current density of 100 mA cm^–2^, using a capillary tube cell setup for visualization. These findings prove that such a thin nano oxide coating is multifunctional in enhancing the cycle life and safety of Li–S batteries.

## Results and discussion

**Fig. 1:**
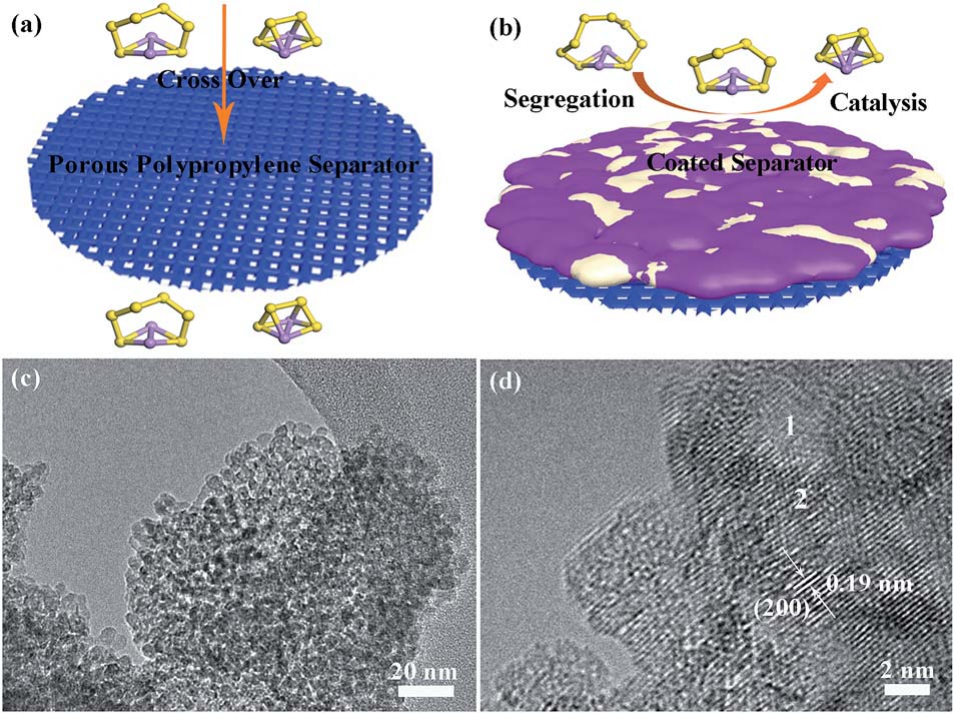
Schematic illustrations of Li–S batteries with (a) a pure polypropylene and (b) a coated separator. (c) Transmission electron microscopy (TEM) and (d) high-resolution transmission electron microscopy (HRTEM) images of the titanium dioxide nanoparticles.

Lithium polysulfides dissolved in the organic electrolyte can easily diffuse through the polypropylene separator and react with the lithium metal anode (Fig. 1a),^22^–^24^ resulting in poor cycling performance.^19^,^20^ Therefore, preventing polysulfide migration by separator modification is a promising strategy to increase the electrochemical performance of Li–S batteries.^25^–^27^ Herein, we prepared titanium dioxide nanoparticles with high Li^+^ conductivity and large specific surface area as a coating on separators (Fig. 1b). The size of the titanium dioxide nanoparticles is ∼3 nm (Fig. 1c and S1†). The amorphous and nanocrystalline phases of the titanium dioxide nanoparticles are shown in area 1 and area 2 (Fig. 1d), respectively. The amorphous phase of TiO_2_ has high lithium ion mobility, which is beneficial for lithium ion diffusion.^28^ The lattice fringes with a distance of 0.19 nm correspond to the (200) plane of anatase TiO_2_ (Fig. S2 and S3†). Moreover, the titanium dioxide nanoparticles show a relatively high specific surface area of 313 m^2^ g^–1^ with a major pore size of 2.5 nm (Fig. S4†), and thus have a large contact area with soluble lithium polysulfides.

**Fig. 2:**
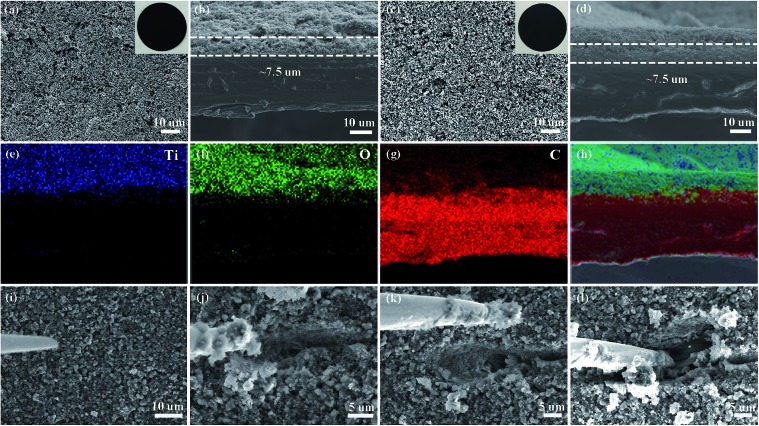
Scanning electron microscopy (SEM) images of the super C65 (C65) separator (a) surface and (b) cross-section, and the titanium dioxide-super C65 (TiO–C65) separator (c) surface and (d) cross-section. The insets in (a) and (c) are digital photographs of the C65 and TiO–C65 separators. (e–h) Energy-dispersive X-ray spectroscopy (EDS) elemental mapping images for the region shown in (d): titanium, oxygen, and carbon. (i–l) Scratching test of the cycled TiO–C65 separator (see Movie S2†) by a nano-manipulator tip shown in SEM images.

The pristine polypropylene separator shows abundant pores on the surface and has a thickness of ∼30 µm (Fig. S5†). After coating with either solely the super C65 or the mixture of titanium dioxide nanoparticles and super C65, the surfaces of these separators become denser (Fig. 2a and c). The thickness of these coating layers is ∼7.5 µm (Fig. 2b and d), and the loadings of the coating material are 0.4 mg cm^–2^ for the C65 separator and 0.7 mg cm^–2^ for the TiO–C65 separator. The coated separator retains its mechanical flexibility, which is important for battery fabrication (Movie S1†). Elemental mapping of the super C65 separator shows that carbon is mainly dispersed on the surface (Fig. S6†). Titanium, oxygen, and carbon are uniformly dispersed on the surface of the TiO–C65 separator (Fig. 2e–h).

To characterize the distribution of sulfur species on the cycled TiO–C65 separator, we performed local probe mechanical tests. In Fig. 2i–l (ESI Movie S2†), we pushed in a sharp tungsten probe from the top of the layer, and then moved it horizontally to execute a scratching test. The particle size on the separator turns out to be bigger compared to that before cycling (Fig. 2c and d), indicating agglomeration bonded by the deposited sulfide (a2 in Scheme 1a). Solid-like sulfur species were confirmed to be deposited on the TiO–C65 coating (Fig. 2i and S12a†). Meanwhile, C65 mixed in the coating (which is in physical contact with the solid cathode) can act as an additional cathode current collector to reuse the lithium polysulfides. When the vertical force applied on the probe is small, we find that there is no extra soft film formed on the surface, unlike in the case of using an acidized carbon nanotube paper on the separator.^12^ Thus, the lithium polysulfides should be dispersed inside the ∼7.5 µm TiO–C65 coating.

**Fig. 3:**
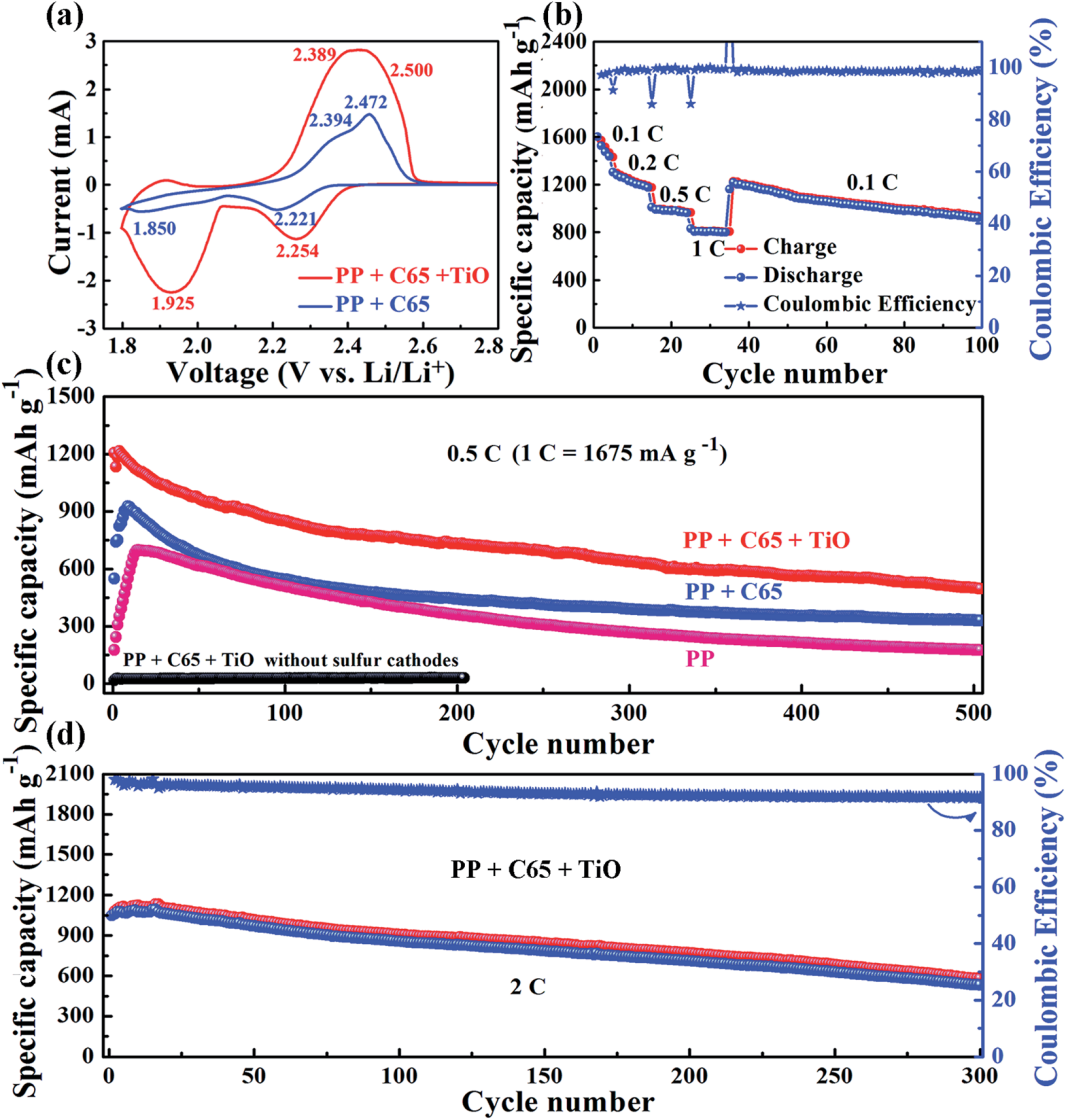
(a) Typical cyclic voltammetry (CV) curves of the sulfur cathode with the TiO–C65 and C65 separator in the 3rd cycle at a scan rate of 0.2 mV s^–1^. (b) Rate capability of the sulfur cathode with the TiO–C65 separator. Cycling performance of sulfur cathodes based on the PP, TiO–C65, and C65 separators at a constant rate of (c) 0.5C and (d) 2C.

From cyclic voltammetry curves, there are two reduction peaks of the S_8_ cathode in Li–S batteries (Fig. 3a).^29^–^31^ The first peak at high voltage corresponds to the open ring reduction of sulfur to soluble lithium polysulfides (Li_2_S_*n*_, 4 ≤ *n* ≤ 8) and the second peak is attributed to the transformation of the lithium polysulfides to insoluble Li_2_S_2_/Li_2_S.^32^ In our Li–S battery test with the C65 separator, the second reduction peak appears in the 3rd cycle (Fig. S7†). However, the TiO–C65 separator Li–S batteries exhibit the second reduction peak after the 1st cycle, and the peak position and shape remain stable from the 2nd cycle on. The cathodic peak positions of the TiO–C65 separator (2.254 and 1.925 V) are larger than the C65 separator (2.221 and 1.850 V), indicating faster redox reaction kinetics. This demonstrates that titanium dioxide nanoparticles have a strong electrocatalytic effect on sulfur reduction (Fig. 3a). There is a small anodic peak at 1.9 V in Li–S batteries with the TiO–C65 separator, corresponding to the lithiation of TiO_2_ (Fig. S8†). Moreover, the overpotential Δ*U* between the anodic peaks and cathodic peaks of the TiO–C65 separator (0.246 and 0.464 V) is smaller than that of the C65 separator (0.251 and 0.544 V), indicating the lower polarization of Li–S batteries with the TiO–C65 separator.

Li–S batteries with the TiO–C65 coated separator show a high specific capacity of 1601 mA h g^–1^ at a current density of 0.1C (1C ≡ 1675 mA g^–1^) and a good rate performance at higher current densities (Fig. 3b). Moreover, the two plateaus in the discharged curve were still evident even up to 1C (Fig. S9†). Typically, the low electronic conductivity of sulfur and the high solubility of lithium polysulfides are associated with the low utilization of sulfur and poor cycling performance of Li–S batteries. In fact, our test with the PP separator shows a low specific capacity of only 175 mA h g^–1^ at a current density of 0.5C (Fig. 3c). On the other hand, the conductive C65 coating on the separator confines the polysulfides within the cathode side, forming a catholyte chamber. Therefore, Li–S batteries with the C65 separator have a higher specific capacity of 550 mA h g^–1^ at the first cycle and maintain a discharged capacity of 332 mA h g^–1^ after 500 cycles at 0.5C. Li–S batteries with the TiO–C65 separator have a high initial capacity of 1206 mA h g^–1^ and a high maintained capacity of 501 mA h g^–1^ after 500 cycles at 0.5C.

The titanium dioxide nanoparticles have a strong catalytic effect and chemical binding with lithium polysulfides, which not only has the potential to increase the utilization but also to improve the rate performance. For example, the charge transfer resistance is clearly reduced (see ESI Fig. S10†). The Li–S batteries with the TiO–C65 coated separator also show a good electrochemical performance at a higher current density of 2C with 1047 mA h g^–1^ at the first cycle and 533 mA h g^–1^ after 300 cycles (Fig. 3d). While the sulfur loading is approximately 2 mg cm^–2^, Li–S batteries with the TiO–C65 separator have an initial capacity of 840 mA h g^–1^ and a high sustained capacity of 556 mA h g^–1^ after 90 cycles at 0.5C (see ESI Fig. S11†). To confirm the ability of the TiO–C65 separator to trap lithium polysulfides, we disassembled the coin cell after the test and performed SEM analysis and found the coexistance of sulfur and titanium (see ESI Fig. S12†). This demonstrates that titanium dioxide nanoparticles can selectively adsorb the sulfur species as a solid-like fouling product.

The trapped sulfur species in the coating can still contribute to the capacity. We constructed a battery cell using the cycled TiO–C65 separator to conduct a cyclic voltammetry scan, and it showed distinct charge/discharge peaks for sulfur (see ESI Fig. S13†), indicating that the coating can act as a second current collector and reuse the lithium polysulfides trapped within. Thereafter, the fouled TiO–C65 coating (after cycling) was applied in a separate H-shaped cell (see ESI Fig. S14†), and showed it could separate the polysulfide containing left side (dark color) from the clear side, demonstrating that the sulfur-containing solid electrolyte formed *in situ* on the TiO–C65 separator could be used to form an isolated catholyte chamber.

To investigate the effects of TiO_2_ on Li_2_S_*n*_ transport and transformation, systematic first-principles calculations were conducted for a Li_2_S_*x*_–graphite (here representing super C65)/TiO_2_ surface system. The optimized lowest-energy geometric structures of Li_2_S_*n*_ (*n* = 1, 2, 4, 6 or 8) are shown in Fig. S15,† which are consistent with other reported works.^33^,^34^ The structures of Li_2_S and Li_2_S_2_ are similar, with sulfur atoms bridging two lithium atoms. Li_2_S_6_ and Li_2_S_8_ show ring-like structures, which can be regarded as a lithium dimer inserted into the S_6_ and S_8_ rings. Li_2_S_4_ has a cage-like structure and is the intermediate structure between the above two structural types.

**Fig. 4:**
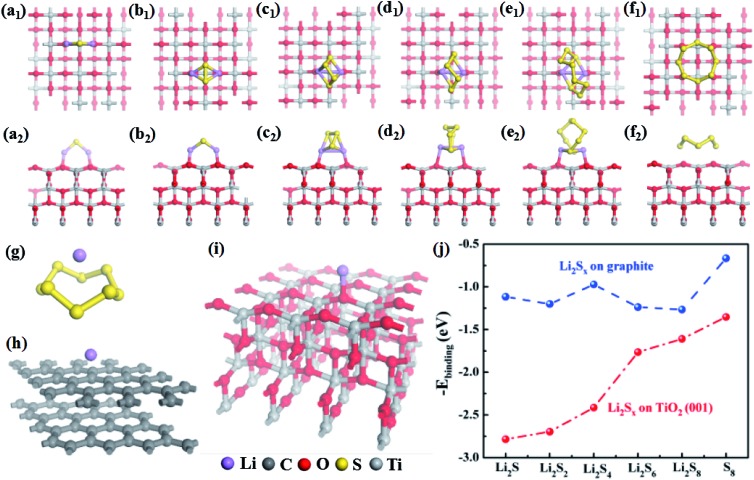
The optimized geometrical structures of (a–e) Li_2_S_*n*_ (*n* = 1, 2, 4, 6, or 8) and (f) S_8_ adsorbed on the TiO_2_ surface. (a_1_–f_1_) The upper and (a_2_–f_2_) lower panels are top and front views of the optimized geometrical structures. The optimized geometrical structures of Li atoms adsorbed on (g) S_8_, (h) graphite, and (i) TiO_2_. The corresponding binding energies are 2.16, 1.68 and 4.45 eV, respectively. (j) The binding energies for Li_2_S_*n*_ (*n* = 1, 2, 4, 6, or 8) and S_8_ adsorbed on the surface of graphite and TiO_2_.

The optimized geometrical models for Li_2_S_*n*_ (*n* = 1, 2, 4, 6, or 8) and S_8_ adsorbed on the TiO_2_ and graphite surfaces are displayed in Fig. 4 and S16,† and the corresponding binding energies are plotted in Fig. 4j. Obviously, the binding energies for Li_2_S_*n*_ adsorbed on the TiO_2_ surface are much larger than those of Li_2_S_*n*_ on the graphite surface. The binding energy of S_8_ on TiO_2_ is 1.35 eV, nearly double that for S_8_ on graphite (0.72 eV), indicating that TiO_2_ can more effectively attract S_8_ molecules. For the graphite and TiO_2_ surfaces, the adsorption energies for Li_2_S_*n*_ are all larger than that for S_8_, which may be related to the larger polarity of Li_2_S_*n*_ compared with S_8_. The binding energies for Li_2_S_*n*_ on graphite are around 1.2 eV except for that for Li_2_S_4_. As shown in Fig. S16,† for Li_2_S and Li_2_S_2_, both lithium atoms are closer to the graphite surface, indicating that the attraction between lithium atoms and graphite is larger than that between sulfur atoms and graphite. On the other hand, the lithium dimers are nearly vertical to the graphite surface for Li_2_S_6_ and Li_2_S_8_. This may be because there is more contact area with graphite when the ring-like Li_2_S_6_ and Li_2_S_8_ are parallel to the graphite surface which maximizes the binding energies. For Li_2_S_4_, the dimer is vertical to the graphite surface. However, it does not have a ring-like structure to maximize the contact area, thus showing a lower binding energy (1.0 eV).

Different from graphite, the binding energies of Li_2_S_*n*_ on TiO_2_ dramatically increase with the decrease of *n* (*n* = 1, 2, 4, 6, or 8) or increase of lithium fraction. As shown in Fig. 4a–f, the lithium atoms are bonded with two adjacent oxygen atoms at the TiO_2_ surface for all of Li_2_S_*n*_ (*n* = 1, 2, 4, 6, or 8). While the distances between the nearest neighbor oxygen atoms are 3.79/3.80 Å, the distances between the lithium atoms in Li_2_S_*n*_ are 3.55, 3.31, 2.85, 2.75, and 2.77 Å, for *n* = 1, 2, 4, 6, and 8, respectively. Our calculation shows that the TiO_2_ surface attracts Li_2_S_*n*_ (*n* = 1, 2, 4, 6, or 8) and S_8_ molecules much more strongly than graphite. With its large surface area, the nano-size TiO_2_ should efficiently adsorb Li_2_S_*n*_ and reduce the shuttling, substantially enhancing the utilization of lithium and sulfur. This explains the expansion of the peak area in the cyclic voltammetry curves (Fig. 3a) and the dramatic improvement of the capacity at higher rates.

Additionally, we compared the abilities of S_8_, graphite and TiO_2_ to attract lithium atoms. The calculated binding energies are 2.16, 1.68 and 4.45 eV, for S_8_, graphite, and TiO_2_, respectively (Fig. 4g, h and i). In the discharge process, lithium ions transport through the separator to react with sulfur atoms. These results give insights into the role of the coating materials on the separator. For example, if there is only carbon (graphite) and no TiO_2_ in the separator, the lithium atoms prefer to adsorb on sulfur but not on graphite, because the binding energy of lithium on the former is larger than that on the latter. Therefore, the effect of carbon alone is expected to be small. Moreover, the obtained lowest-energy Li–S_8_ adsorption configuration has the lithium atom located above the center of the S_8_ molecule (Fig. 4g), and it requires breaking the S_8_ ring (overcoming an energy barrier of about 1.5 eV)^35^ to form the most stable Li_2_S_8_ structure (Fig. S15e†). In contrast, the binding energy for the lithium atom on TiO_2_ is about twice as large as that on S_8_, which allows TiO_2_ to attract lithium atoms in addition to S_8_ molecules (as discussed above). This leads to the aggregation of lithium atoms and S_8_ molecules on the TiO_2_ surface. Additionally, each lithium atom releases about 4 eV when binding with TiO_2_, which can help the nearby S_8_ to overcome the barrier to open the ring and form the Li_2_S_8_ structure. TiO_2_ thus provides an effective electrocatalytic surface by stabilizing the reaction intermediates, and enhancing the rate of reaction between them. Furthermore, it is known that once the size of TiO_2_ is smaller than 10 nm, it can become electrically conductive.^36^ This electrocatalytic effect for the Li_2_S_*n*_ transformations explains the peak shifts in the cyclic voltammetry curves (Fig. 3a) and the reduction of the activation period (Fig. 3c).

With the above electrochemical tests and theoretical calculations, we confirm that our TiO–C65 coated membrane can trap lithium polysulfides to improve the performance of Li–S batteries. The nanoparticle coating may exhibit an additional benefit, which is to prevent lithium dendrite penetration. To test the capability of the coated separator to block dendritic lithium penetration, we devised a capillary cell to visualize the electrodeposition process. The capillary cell is filled with the liquid electrolyte, where a piece of lithium metal electrode is stripped and the lithium ions are simultaneously electrodeposited onto an enamelled copper wire with the end wrapped by the separators. The detail of the experimental setup is shown in the ESI Fig. S17.† The diameter of the copper wire is 0.04 cm. The capillary tube batteries are discharged at a high current density of 100 mA cm^–2^ to promote the dendritic growth of lithium metal. Lithium dendrites begin to emerge from the polypropylene separator at 50 s (ESI Movie S3†), while no lithium penetration was observed in the cell using our TiO–C65 separator even after 850 s (ESI Movie S4†). The results indicate that the TiO–C65 coating can act as a multifunctional barrier to prevent the lithium dendrites from penetrating the separator, as well as preventing the cross-over of lithium polysulfides.

## Conclusions

In summary, we have developed a multi-functional titanium dioxide–super C65 modified separator for Li–S batteries that enables a high specific capacity, stable cycling performance at high rates, and improved safety. Li–S batteries with the TiO–C65 separator show a high initial capacity of 1206 mA h g^–1^ and maintain a high specific capacity of 501 mA h g^–1^ after 500 cycles at 0.5C. The electrochemical results and theoretical simulation demonstrate that titanium dioxide nanoparticles have a strong catalytic effect and chemical binding with lithium polysulfides. Therefore, the effect of the TiO–C65 separator is assigned to (a1) surface segregation and catalysis, and also the partial effect of (a2) sealing by the solid electrolyte formed *in situ*. The results of this work indicate that thin coating materials with high conductivity and a large surface area on the separator can increase the utilization of lithium polysulfides, allow the fast diffusion of lithium ions, and decrease the migration of lithium polysulfides to the lithium metal anode. Additionally, our titanium dioxide nanoparticle–super C65 separator with a strong dendrite blocking ability can be used in applications beyond Li–S batteries such as lithium/sodium metal batteries, and contributes to the development of high-performance and safe energy storage devices.

## Supplementary Material

Supplementary movie

Supplementary movie

Supplementary movie

Supplementary movie

Supplementary information
