# Supplementary material for: A thin multifunctional coating on a separator improves the cyclability and safety of lithium sulfur batteries
Source: Chem Sci. 2017 Jul 17;8(9):6619–25. doi: 10.1039/c7sc01961k (PMC5676610; doi:10.1039/c7sc01961k)
Supplement: Supplementary file 5 [file SC-008-C7SC01961K-s005.pdf]

Electronic Supplementary Material (ESI) for Chemical Science.
This journal is © The Royal Society of Chemistry 2017

Supporting Information

Thin Multifunctional Coating on Separator Improves Cyclability and Safety of Lithium Sulfur Battery

Guiyin Xu,[a,b] Qing-bo Yan,[b,c] Shitong Wang,[b] Akihiro Kushima,[b] Peng Bai,[d] Kai Liu,[b]

Xiaogang Zhang,[a,] Zilong Tang,[e] Ju Li[b,f,]**

[a] Jiangsu Key Laboratory of Material and Technology for Energy Conversion, College of Material Science and Engineering, Nanjing University of Aeronautics and Astronautics, Nanjing 210016, China

[b] Department of Nuclear Science and Engineering, Massachusetts Institute of Technology, Cambridge, Massachusetts 02139, USA

[c] College of Materials Science and Opto-Electronic Technology, University of Chinese Academy of Sciences, Beijing 100049, China

[d] Department of Chemical Engineering, Massachusetts Institute of Technology, Cambridge, Massachusetts 02139, USA

[e] State Key Lab of New Ceramics and Fine Processing, School of Materials Science and Engineering, Tsinghua University, Beijing 100084, PR China

[f] Department of Materials Science and Engineering, Massachusetts Institute of Technology, Cambridge, Massachusetts 02139, USA

Experimental Section

Preparation of Titanium Dioxide Nanoparticles. Titanium dioxide nanoparticles were synthesized *via* a simple hydrolysis process of $TiOSO_4 \cdot 2H_2O$ aqueous solutions. The typical procedure was as follows: 0.25 M $TiOSO_4 \cdot 2H_2O$ aqueous solutions were prepared and placed in a 100 mL Teflon-lined autoclave. Then it was heated to 150 °C for 5-120 min. After the hydrothermal reaction, the products were washed by distilled water thoroughly, followed by heat treatment in vacuum to obtain white titanium dioxide powder.

Preparation of the C65 Separator and the TiO-C65 Separator. The 70 wt% titanium dioxide nanoparticles, 20 wt% timcal super C65, and 10 wt% polyvinylidene fluoride (PVDF) were stirred for 12 h in N-methyl pyrrolidinone (NMP) and then were coated on the polypropylene separator by the doctor blade. The separator was dried at 60 °C overnight and the TiO-C65 separator was obtained after the material was cooled to room temperature. The C65 separator consisted of 90 wt% timcal super C65 and 10 wt% PVDF on the polypropylene separator. The other procedures are similar with the TiO-C65 separator.

Characterization. X-ray diffraction (XRD) patterns were measured on a Bruker-AXS D8 DISCOVER. Copper Kα line was used as a radiation source with λ=0.15406 nm. Field emission scanning electron microscopy (FESEM) and transmission electron microscopy (TEM) measurements were carried out with JEOL JSM-6380LV FE-SEM and FEI TECNAI-20, respectively. Scanning transmission electronic microscope (STEM) was performed on a Tecnai G2 F30. The X-ray photoelectron spectroscopy (XPS) analysis was performed on a Perkin-Elmer PHI 550 spectrometer with Al Kα (1486.6 eV) as the X-ray source. The N_2 adsorption/desorption tests were determined by Brunauer-Emmett-Teller (BET) measurements using an ASAP-2010 surface area analyzer. The pore size distribution (PSD) was derived from the desorption branch of the isotherm with the Barrett-Joyner-Halenda (BJH) method.

Electrochemical Characterization. Electrochemical characterization was carried out by galvanostatic cycling in CR2032-type coin cells. The working electrodes were prepared by a slurry coating procedure. The slurry consisted of 60 wt% commercial sulfur, 30 wt% timcal super C65 and 10 wt% polyvinylidene fluoride (PVDF) dissolved in N-methyl pyrrolidinone (NMP), and was uniformly spread on an aluminium foil current collector. Finally, the electrode was dried at 60 °C for 24 h. Each current collector contained *ca* 2.0 mg cm^{-2} material (with *ca* 1.2 mg cm^{-2} S). Test cells were assembled in an argon-filled glove box using the lithium metal anode and the polypropylene separator or the TiO-C65 separator or the C65 separator. The electrolyte was 1 mol L^{-1} LiTFSI and 0.2 mol L^{-1} $LiNO_3$ in a mixed solvent of 1, 3-dioxolane (DOL) and 1, 2-dimethoxyethane (DME) with a volume ratio of 1:1. The amount of the electrolyte used in the cell is ~10 uL. The coin cells were galvanostatically charged-discharged at different current densities between 1.8 and 2.8 V (*vs.* Li/Li^+) using a CR2032 coin cell test instrument (LAND Electronic Co.). The cyclic

voltammetry (CV) measurement was conducted with a Gamry electrochemical workstation at a scan rate of 0.2 mV s^{-1} in the voltage range of 1.8 to 2.8 V (*vs.* Li/Li^{+}). Electrochemical impedance spectroscopy (EIS) was measured in the frequency range of 100 kHz-10 mHz with an amplitude of 5 mV.

Computational Section. The calculations were performed using Vienna ab initio simulation package (VASP),[1] which was based on density functional theory (DFT)[2] and the projected augmented wave (PAW) method.[3] Generalized gradient approximation (GGA) in the form of Perdew-Burke-Ernzerhof (PBE)[4] was chosen as exchange correlation potential. The DFT-TS method[5] was adopted to take into account the van der Waals interactions. The kinetic energy cutoff for plane wave functions was set to 500 eV. All structures were fully optimized and the maximum force on each atom was less than 0.01 eV $Å^{-1}$. Supercells containing four atomic layers (the bottom bilayer was fixed during optimization) and a vacuum spacing larger than 15 Å was used to model the graphite and TiO_2 (001) surface. Various initial adsorption configurations were considered, and the most stable optimized configurations were used to discuss the binding between Li_2S_n and graphite or TiO_2 surface. The binding energies (E_b) are defined as the difference between the total energy (E_{tot}) of Li_2S_n-surface adsorption systems, and the energy sum of Li_2S_x and graphite or TiO_2 surface:

$$E_b = (E_{Li_2S_n} + E_{surface}) - E_{tot}$$

Under the above definition, the calculated positive or negative binding energies represent attraction and repel interactions between Li_2S_n and graphite or TiO_2 surface, respectively. The more positive binding energy corresponds to the stronger adsorption.

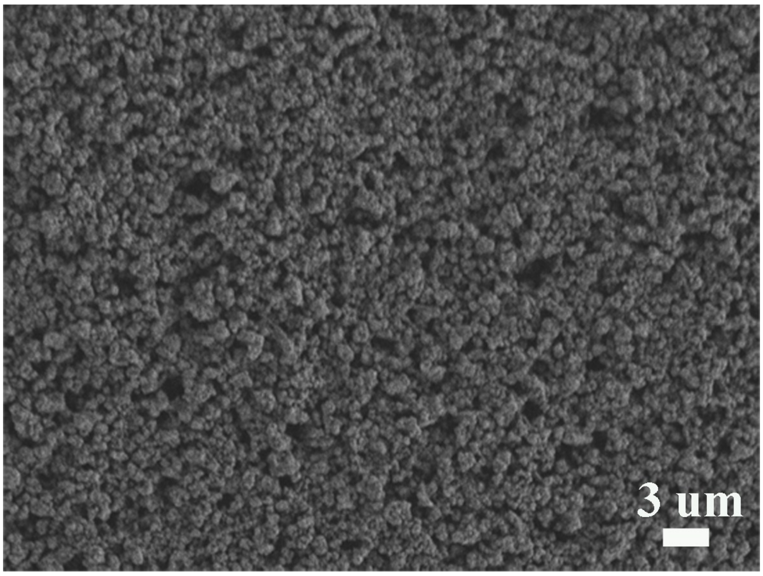

Figure S1. Scanning electron microscopy (SEM) image of titanium dioxide nanoparticles.

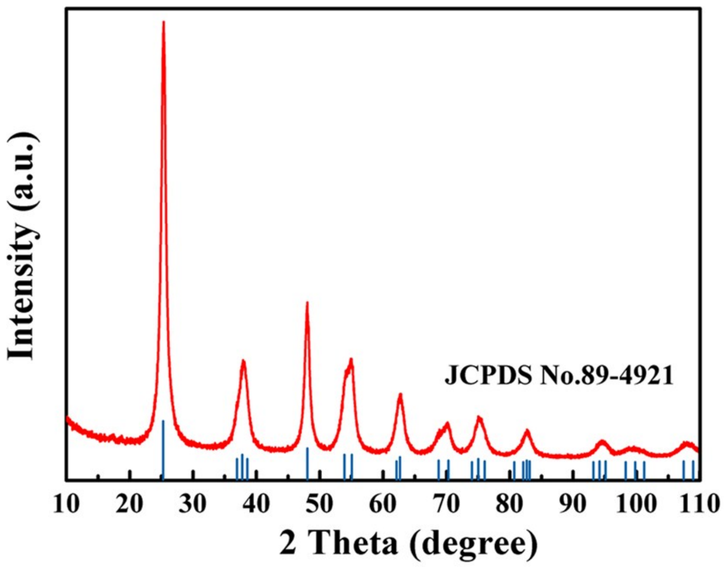

Figure S2. XRD pattern of titanium dioxide nanoparticles.

The XRD pattern of the as-prepared sample shows the diffraction peaks consistent with anatase TiO_2 (JCPDS No. 89-4921) with the planes indexed. The very weak intensity suggests a poorly crystallized structure and possible containing of amorphous phase.

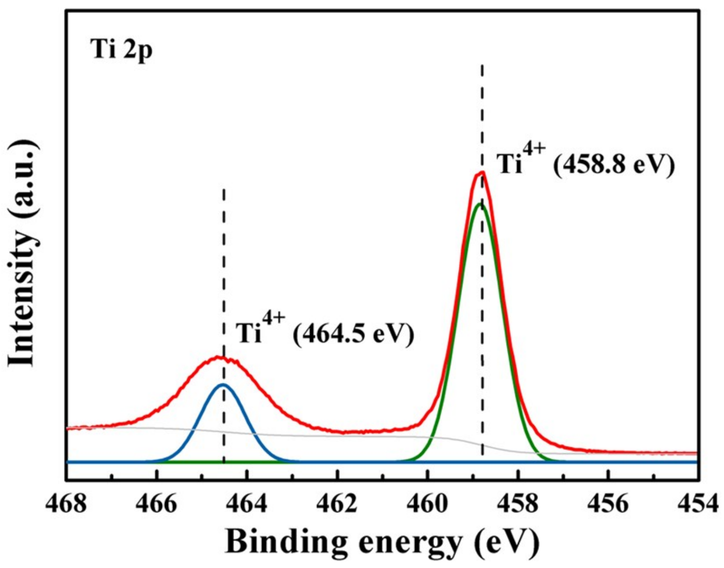

Figure S3. Ti 2*p* XPS spectra of titanium dioxide nanoparticles (Red: observed data; Gray: background; Blue and green: fitting data).

Ti 2*p* X-ray photoelectron spectroscopy (XPS) spectra shows similar features of Ti^{4+} ions due to the Ti $2p_{3/2}$ (≈458.8 eV), Ti $2p_{1/2}$ (≈464.5 eV),[6] and no Ti^{3+} signal is detected.[7-9]

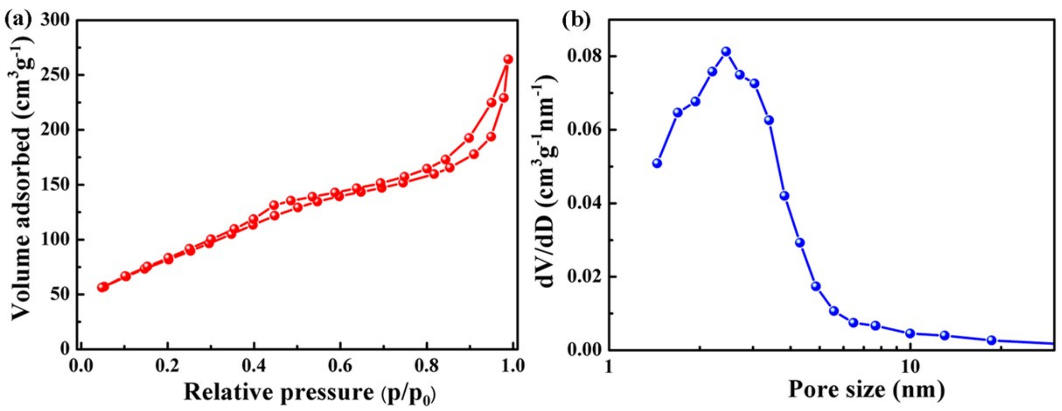

Figure S4. (a) N_2 adsorption/desorption isotherms at 77 K and (b) pore size distribution (PSD) curves of titanium dioxide nanoparticles. PSD is calculated using the Barrett-Joyner-Halenda (BJH) method.

Titanium dioxide nanoparticles show a relatively high surface area of 313 m^2 g^{-1} with a major pore size of 2.5 nm.

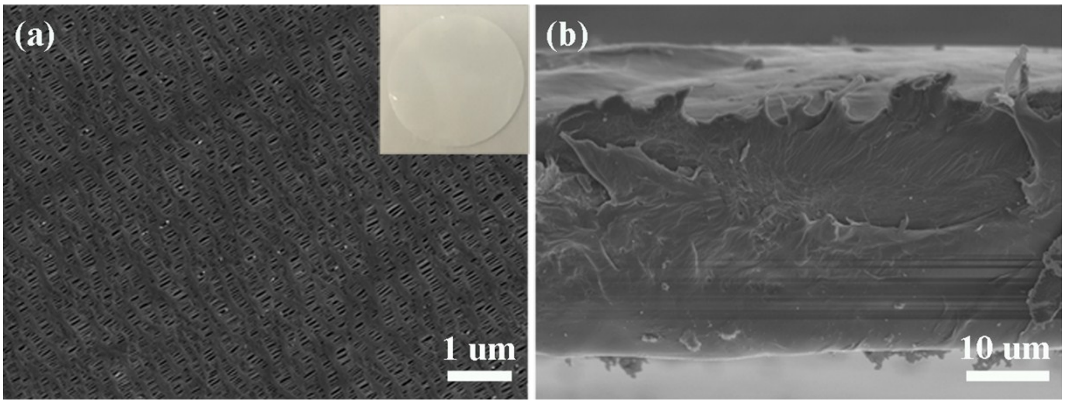

Figure S5. SEM images of the polypropylene (PP) separator (a) surface and (b) cross-section. The inset in **Figure S5a** is the digital photograph of the PP separator.

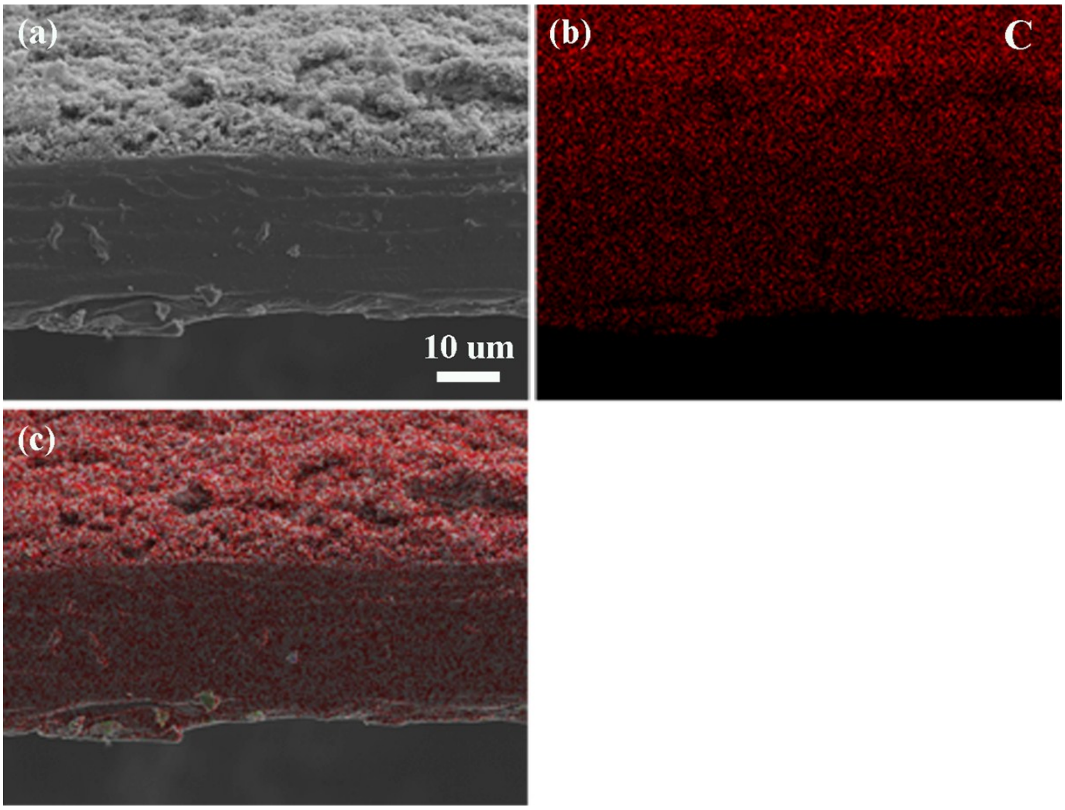

Figure S6. (a) SEM image of the C65 separator and (b) corresponding elemental mapping image of carbon.

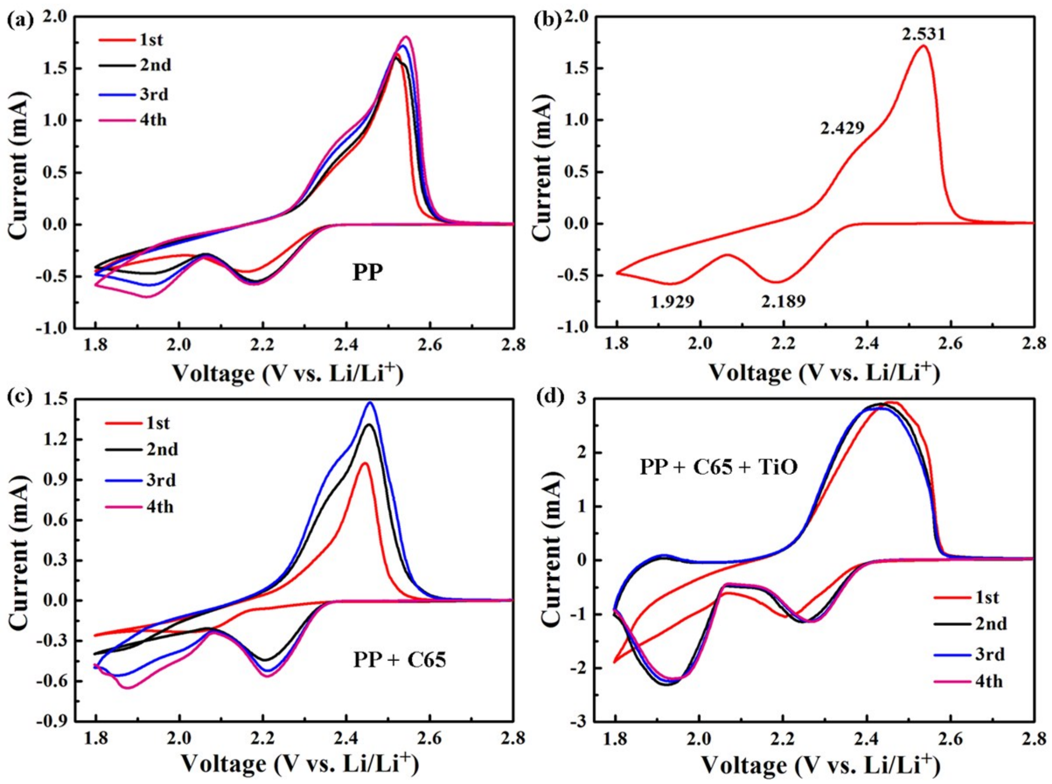

Figure S7. Typical cyclic voltammetry (CV) curves of the sulfur cathode with (a, b) the PP separator, (c) the C65 and (d) TiO-C65 coated separator before cycling at a scan rate of 0.2 mV s^{-1}. (b) CV curve of the sulfur cathode with the PP separator in the 3rd cycle. The cathodic peak position of the PP separator (2.189 V) is smaller than that of the TiO-C65 separator (2.254 V) and the C65 separator (2.221 V). Moreover, the overpotential ΔU between the anodic peak and cathodic peak of the PP separator (0.342 V) is larger than that of the TiO-C65 separator (0.246 V) and the C65 separator (0.251 V).

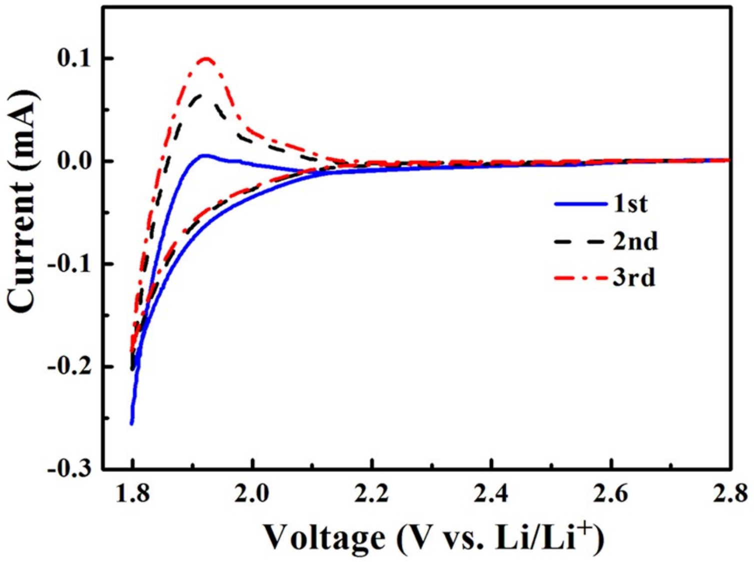

Figure S8. Typical cyclic voltammetry (CV) curves of the TiO-C65 coated separator without sulfur cathode before cycling at a scan rate of 0.2 mV s^{-1}.

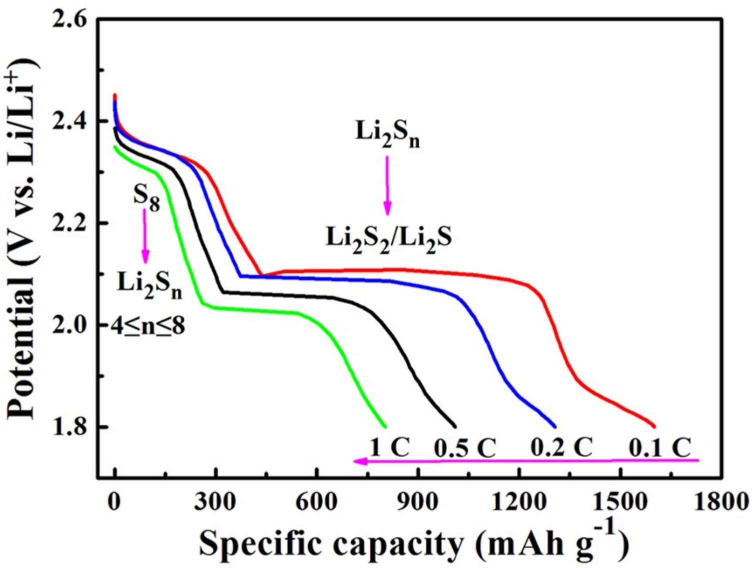

Figure S9. Galvanostatic discharge profiles at different rates of the sulfur cathode with the TiO-C65 coated separator.

The two plateaus correspond to the reduction peaks in **Figure 3a**. The first plateau in high potential corresponds to the open ring reduction of S_8 to soluble lithium polysulfides (Li_2S_n, $4 \leq n \leq 8$) and the second plateau is attributed to the transformation of the lithium polysulfides to insoluble Li_2S_2/Li_2S.

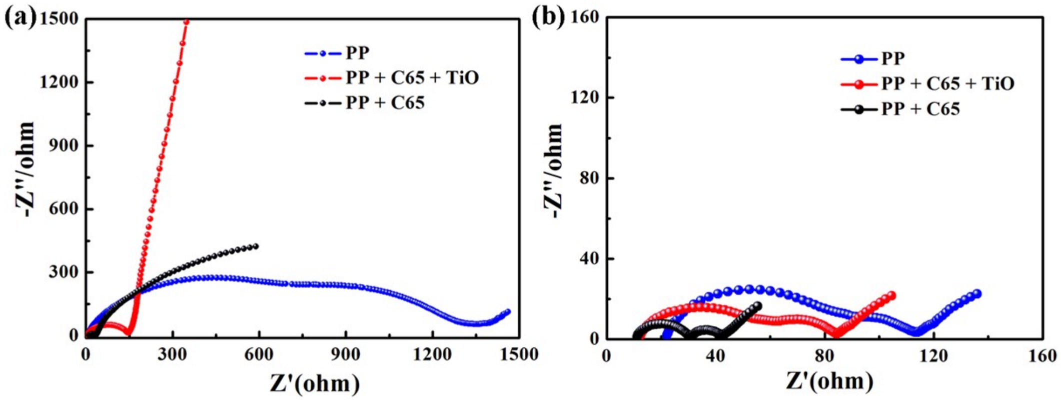

Figure S10. Impedance plots of sulfur cathodes based on the PP separator, TiO-C65 coated separator, and C65 coated separator (a) before cycling and (b) after 100 cycles at at a current density of 0.5 C.

The impedance plots consist of a straight line in the low frequency region corresponding to the ion diffusion (the Warburg impedance, W) and a semicircle in the high frequency region relating to the interface charge-transfer process (the charge transfer resistance, R_{ct}) before cycling.[10] The charge transfer resistance of the TiO-C65 separator and C65 separator is smaller than that of the PP separator (**Figure S10a**). For the sulfur cathodes after 100 cycles at a current density of 0.5 C, the impedance plots exhibit two depressed semicircles and a sloping line (**Figure S10b**). The charge transfer resistance of the TiO-C65 separator and C65 separator is also smaller than that of the PP separator.

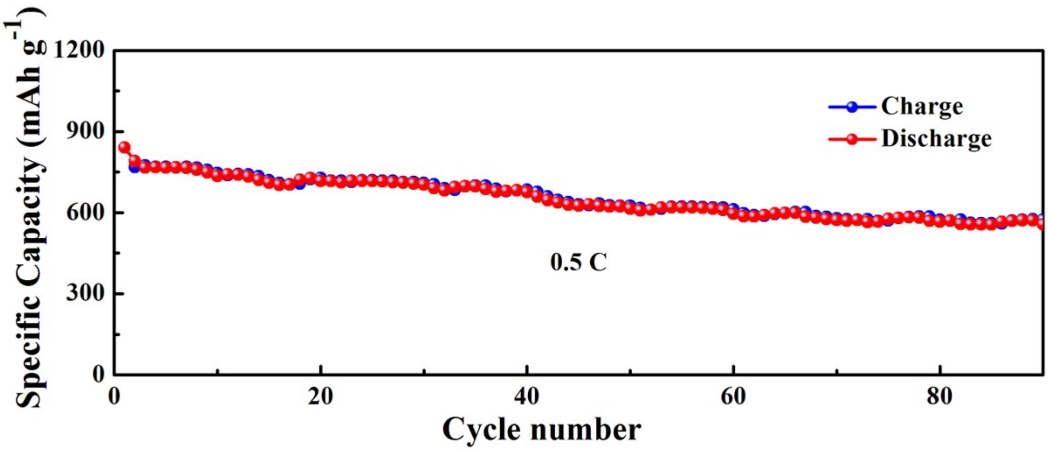

Figure S11. Cycling performance of the sulfur cathode based on the TiO-C65 separator at a current density of 0.5 C. The sulfur loading is *ca* 2 mg cm^{-2}.

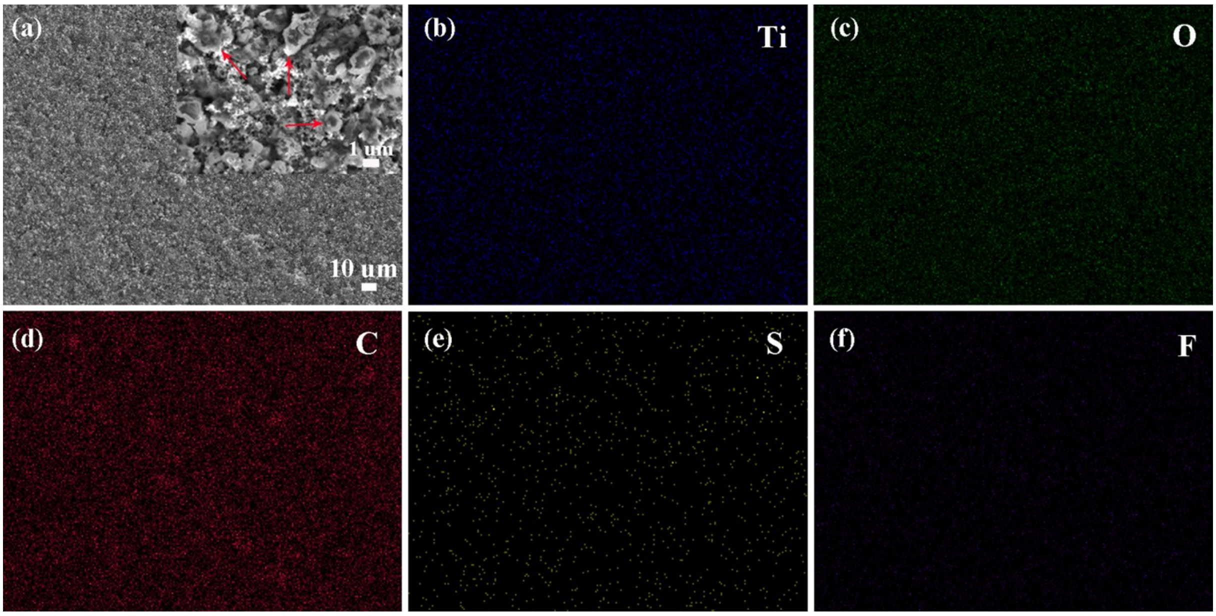

Figure S12. SEM and energy-dispersive X-ray spectroscopy (EDS) characterization of the TiO-C65 coated separator after 50 cycles at 0.5 C (charged state): (a) SEM images of the cycled TiO-C65 separator. The sulfur active material deposition is shown in the red arrow. (b-f) EDS elemental maps for the region shown in (a): (b) titanium, (c) oxygen, (d) carbon, (e) sulfur, and (f) fluorine.

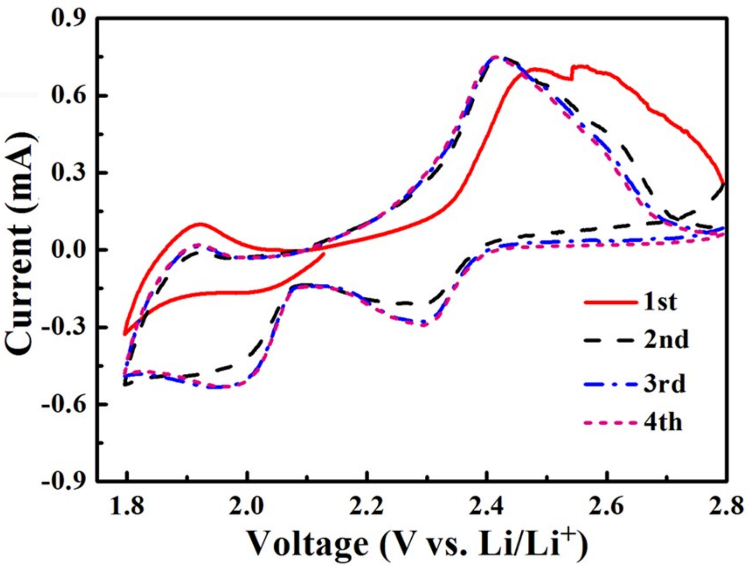

Figure S13. Typical CV curves of the cycled TiO-C65 separator (after 50 cycles at 0.5 C) without sulfur cathode at a scan rate of 0.2 mV s^{-1}.

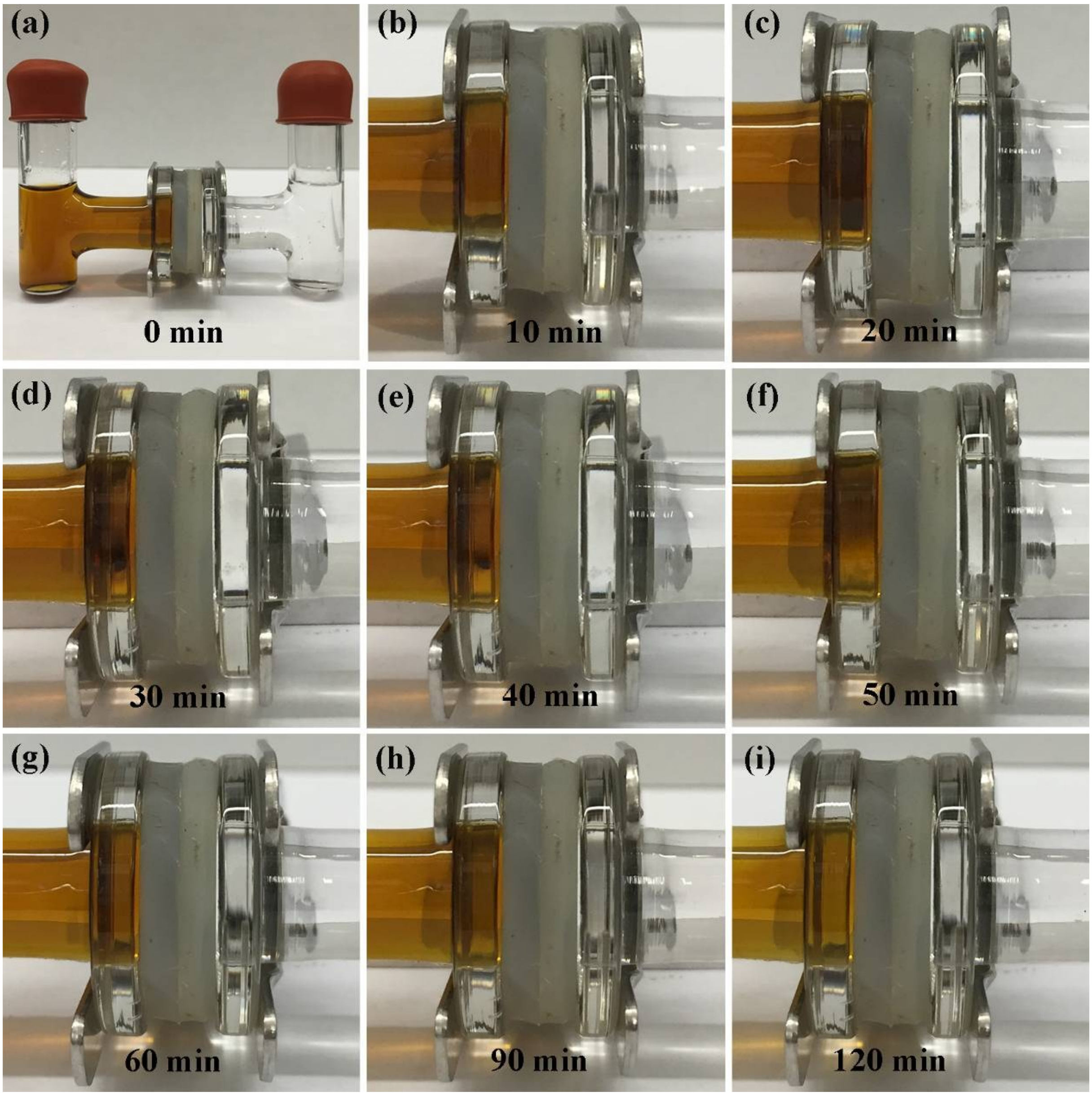

Figure S14. Digital photographs of the H-cell with the cycled TiO-C65 separator at different times.

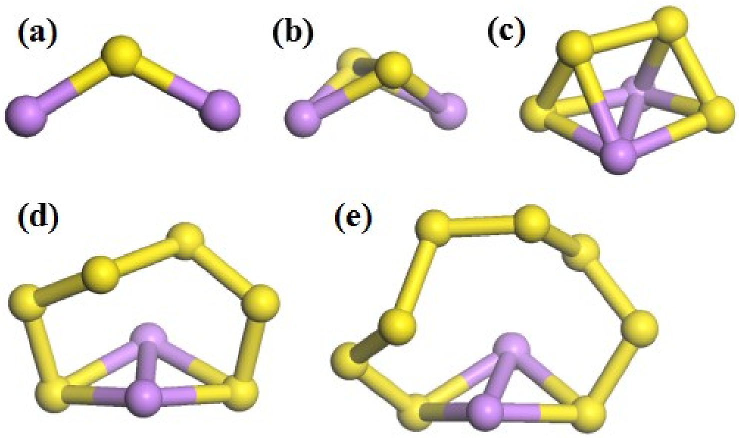

Figure S15. The optimized geometrical structures of Li_2S_n (n=1, 2, 4, 6, 8). Yellow is sulfur atom while purple is lithium atom.

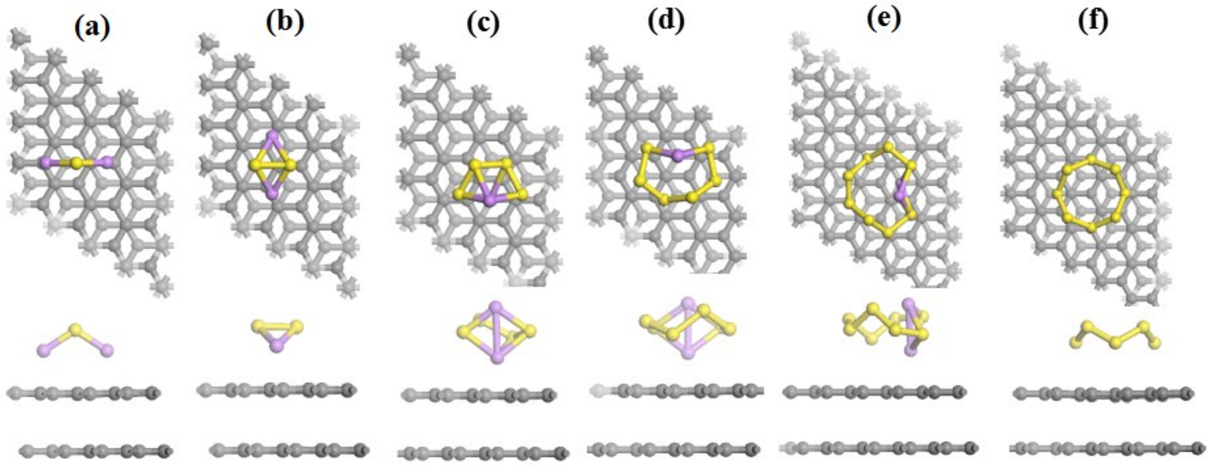

Figure S16. The optimized geometrical structures of (a-e) Li_2S_n (n=1, 2, 4, 6, 8) and (f) S_8 adsorbed on graphite. The upper and lower panels are top and front views, respectively. Only the top two layers of graphite are shown here.

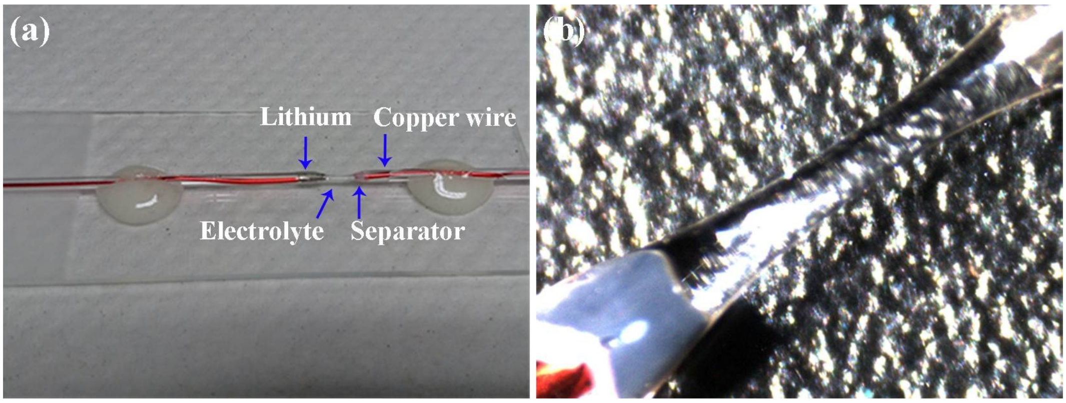

Figure S17. (a) Digital photographs of the capillary tube battery and (b) local enlarged image in the center of a.

Movie S1. The appearance and flexibility of the TiO-C65 separator.

Movie S2. The tension of the cycled TiO-C65 separator after 50 cycles at 0.5 C (Played at x5 original speed).

Movie S3. The capillary tube battery based on the polypropylene separator (Played at x5 original speed).

Movie S4. The capillary tube battery based on the TiO-C65 separator (Played at x5 original speed).

References

1 G. Kresse and J. Furthmüller, *Phys. Rev. B*, 1996, **54**, 11169-11186.

2 P. Hohenberg and W. Kohn, *Phys. Rev.*, 1964, **136**, B864-B871.

3 G. Kresse and D. Joubert, *Phys. Rev. B*, 1999, **59**, 1758-1775.

4 J. P. Perdew, K. Burke and M. Ernzerhof, *Phys. Rev. Lett.*, 1996, **77**, 3865-3868.

5 A. Tkatchenko and M. Scheffler, *Phys. Rev. Lett.*, 2009, **102**, 073005.

6 J. Chen, W. Song, H. Hou, Y. Zhang, M. Jing, X. Jia and X. Ji, *Adv. Funct. Mater.*, 2015, **25**, 6793-6801.

7 L. Shen, E. Uchaker, X. Zhang and G. Cao, *Adv. Mater.,* 2012, **24**, 6502-6506.

8 A. Naldoni, M. Allieta, S. Santangelo, M. Marelli, F. Fabbri, S. Cappelli, C. L. Bianchi, R. Psaro and V. Dal Santo, *J. Am. Chem. Soc.* 2012, **134**, 7600-7603.

9 X. Chen, L. Liu, P. Y. Yu and S. S. Mao, *Science* 2011, **331**, 746-750.

10 G. Xu, J. Han, B. Ding, P. Nie, J. Pan, H. Dou, H. Li and X. Zhang, *Green Chem.* 2015, **17**, 1668-1674.
